# Erratum to: A Two-Locus System with Strong Epistasis Underlies Rapid Parasite-Mediated Evolution of Host Resistance

**DOI:** 10.1093/molbev/msab076

**Published:** 2021-04-12

**Authors:** Camille Ameline, Yann Bourgeois, Felix Vögtli, Eevi Savola, Jason Andras, Jan Engelstädter, Dieter Ebert

*Mol. Biol. Evol.* doi: 10.1093/molbev/msaa311

In the originally published version of this manuscript, amendments were inadvertently made without author approval prior to publishing, making key data incomprehensible to the reader. The errors have now been corrected.

