# Supplementary material for: A Two-Locus System with Strong Epistasis Underlies Rapid Parasite-Mediated Evolution of Host Resistance
Source: Mol Biol Evol. 2020 Dec 1;38(4):1512–28. doi: 10.1093/molbev/msaa311 (PMC8042741; doi:10.1093/molbev/msaa311)
Supplement: msaa311_Supplementary_Data [file msaa311_supplementary_data.pdf]

Supplementary Material – A two-locus system with strong epistasis underlies rapid parasite-mediated evolution of host resistance

Camille Ameline[1], Yann Bourgeois[1,2], Felix Vögtli[1], Eevi Savola[1,3], Jason Andras[1,4], Jan Engelstädter[5] and Dieter Ebert[1]

[1]Department of Environmental Sciences, Zoology, University of Basel, Vesalgasse 1, 4051 Basel, Switzerland
[2] School of Biological Sciences, King Henry Building, King Henry I Street, University of Portsmouth, Portsmouth, PO1 2DY, UK
[3]Institute of Evolutionary Biology, University of Edinburgh Ashworth Laboratories Charlotte Auerbach Road Edinburgh EH9 3FL
[4]Department of Biological Sciences, Clapp Laboratory, Mount Holyoke College, South Hadley, MA, USA
[5]School of Biological Sciences, The University of Queensland, Brisbane, Australia

Corresponding author: Camille Ameline, cameline8@gmail.com

Overview

Section in main manuscript	Element	Description	Page
**Results**			
Parasite-mediated selection explains phenotypic dynamics			
Monitoring	Fig. S1	*Pasteuria* genotypes	p. 2
Linking resistance phenotypes to genotypes	Fig. S2	ABC genetic model	p. 3
Genomic regions of resistance to the parasite	Table S1 and S2	E locus description	p. 4
Genetic model of resistance inheritance	Table S3	GWAS genotypes	p. 5
	Tables S4 to S15	Selfing results	p. 6-11
Linking the genomic regions and the genetic model of resistance	Tables S16 to S18	Markers linkage	p. 12-14
**Methods**			
Assessment of resistotype segregation	Table S19	Selfing methods	p. 15
Predictions of segregation patterns	Documents S1 and S2	Peas program	p. 16-20
Linking the phenotype to the genotype	Tables S20 and S21	Genetic markers	p. 21-23
	References		p. 24

Results

Parasite-mediated selection explains phenotypic dynamics

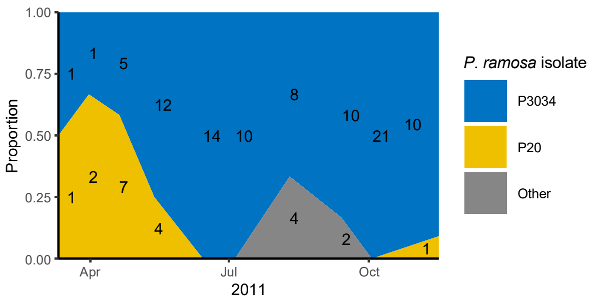

Fig. S1. *Pasteuria ramosa* genotypes frequency over time during the 2011 active season of *Daphnia magna.* Infected animals were collected throughout the season, and the genotype of the parasite was determined using microsatellite markers following the protocol described in (Andras and Ebert 2013).A total of 113 infected *D. magna* individuals were sampled throughout the season and their parasite genotype was assessed. The P20 genotype represents about 50% of the parasite diversity among infected hosts at the beginning of the season, a proportion that then drops off during the epidemics. The P3034 genotype represents the most diversity of the parasite population among infected hosts but was not used in this study to score host resistotype (resistance phenotype). Numbers represent the amount of *D. magna* individuals sampled on each collection date.

Linking resistance phenotypes to genotypes

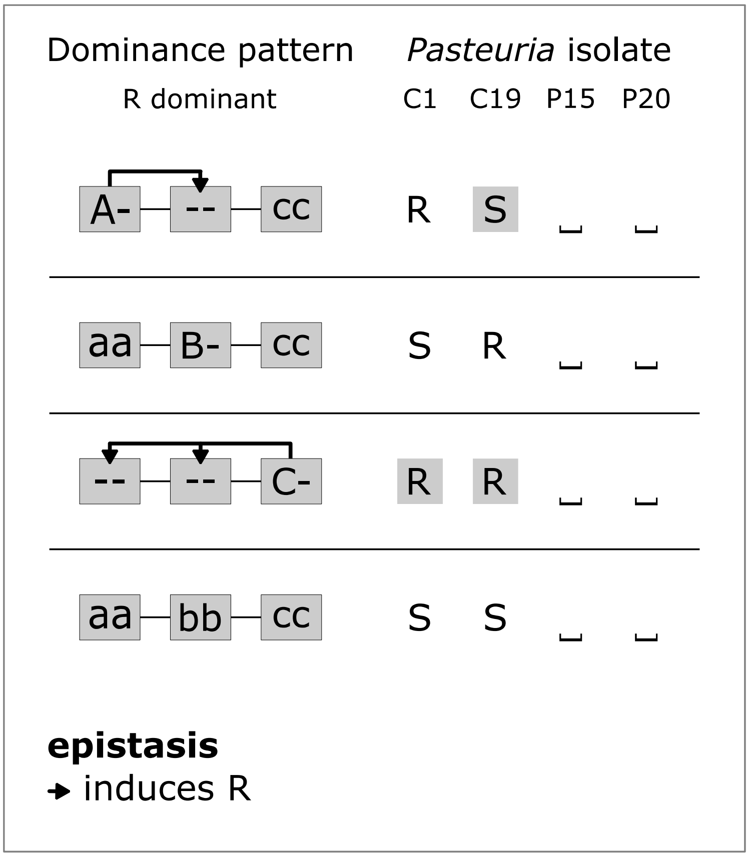

Fig. S2. Genetic model of resistance inheritance at the ABC-cluster in the *D. magna* - *P. ramosa* system. Resistance is dominant at the A, B and C loci. The dominant allele at the A locus confers resistance to C1 and susceptibility to C19, regardless of the genotype at the B locus (epistasis). The dominant allele at the B locus confers resistance to C19, in the right genetic background, i.e. when an individual is double recessive at the A locus ("aa" genotype). The dominant allele at the C locus acts epistatically on the A and B loci and confers resistance to C1 and C19, regardless of the genotype at the A and B loci. Figure adapted from (Metzger et al. 2016).

Table S1. Genomic description of the E locus. The region displaying the most markers discriminating between ␣␣␣R and ␣␣␣S encompassed 22 scaffolds and one contig on version 2.4 of the *Daphnia magna* reference genome (Routtu et al. 2014), with a cumulated length of 3101076 bp. The strongest signals of association were found on scaffolds 2167 and 2560. Scaffolds are ordered following the genetic map of (Dukić et al. 2016).

Scaffold	Length (bp)
**scaffold02167**	358328
contig71262	772
scaffold00149	66134
scaffold00715	344834
scaffold01200	81121
scaffold01244	6526
scaffold01745	65595
scaffold01839	55648
scaffold01865	165487
scaffold02315	259660
scaffold02388	172183
scaffold02489	70866
**scaffold02560**	89801
scaffold02708	22268
scaffold02913	44097
scaffold02970	253762
scaffold02993	133684
scaffold03031	266621
scaffold03154	80879
scaffold03356	21979
scaffold02031	8800
scaffold02207	115428
scaffold00872	416603

Table S2. We found 485 genes on all associated scaffolds. Scaffolds 2167 and 2560 harbored 82 candidate genes. Some of these genes had annotations similar to genes identified in a previous study of the ABC locus (Bento et al. 2017), with a glucosyltransferase found on scaffold 2167. Three other sugar transferases (galactosyltransferases) were identified on the 22 associated scaffolds, two of them found on scaffold 2560.

Scaffold	Start	End	Name
scaffold02489	49633	55225	udp-galactose:n-acetylgalactosamine-alpha-r beta galactosyltransferase/ARP2_G2977
**scaffold02560**	1296	4007	udp-galactose:n-acetylgalactosamine-alpha-r beta galactosyltransferase/ARP2_G2977
**scaffold02560**	4186	14123	udp-galactose:n-acetylgalactosamine-alpha-r beta galactosyltransferase/ARP2_G2977
**scaffold02167**	334036	335960	glucosyl/glucuronosyl tr_G27

Table S3. List of *D. magna* genotypes used for the association analysis. Genotypes whose name starts with "CH-H" were collected directly from the pond between 2010 and 2014. Names starting with "t" depict animals hatched in the laboratory from resting eggs collected in early 2014, before the natural hatching of the *D. magna* population.

Clone	Resistotype C1, C19, P15, P20	Resistotype C1, C19, _ , P20	C-locus genotype	E-locus genotype
CH-H-1769	RRRR	RR_R	Cc	ee
CH-H-2029	RRRR	RR_R	Cc	ee
CH-H-2299	RRRR	RR_R	CC	ee
t1_10.3_2	RRRR	RR_R	CC	ee
t2_14.3_10	RRRR	RR_R	CC	ee
CH-H-1	RRSR	RR_R	Cc	ee
t1_10.3_4	RRSR	RR_R	Cc	ee
t3_10.3_12	RRSR	RR_R	Cc	unsure
t3_14.3_4	RRSR	RR_R	CC	ee
t3_14.3_5	RRSR	RR_R	Cc	ee
t3_31.3_1	RRSR	RR_R	Cc	ee
t4_10.3_11	RRSR	RR_R	CC	ee
t4_10.3_12	RRSR	RR_R	Cc	ee
t4_10.3_2	RRSR	RR_R	Cc	ee
t5_10.3_5	RRSR	RR_R	CC	ee
t5_20.3_1	RRSR	RR_R	CC	ee
t2_17.3_1	RRSS	RR_S	Cc	Ee
t2_17.3_4	RRSS	RR_S	Cc	Ee
t2_17.3_5	RRSS	RR_S	Cc	EE
t3_12.3_1	RRSS	RR_S	CC	Ee
t3_31.3_3	RRSS	RR_S	CC	EE
t4_13.3_10	RRSS	RR_S	Cc	EE
t4_17.3_8	RRSS	RR_S	Cc	Ee
t4_17.3_9	RRSS	RR_S	CC	Ee
t5_12.3_3	RRSS	RR_S	CC	Ee
t5_12.3_4	RRSS	RR_S	Cc	Ee
CH-H-16	SSSS	SS_S	cc	Ee
t2_12.3_10	SSSS	SS_S	cc	Ee
t2_12.3_11	SSSS	SS_S	cc	ee
t2_12.3_12	SSSS	SS_S	cc	ee
t2_14.3_9	SSSS	SS_S	cc	EE
t3_12.3_6	SSSS	SS_S	cc	ee
t3_12.3_8	SSSS	SS_S	cc	EE
t3_13.3_5	SSSS	SS_S	cc	Ee
t3_18.3_3	SSSS	SS_S	cc	Ee
t4_12.3_3	SSSS	SS_S	cc	EE
t4_7.3_4	SSSS	SS_S	cc	Ee

Tables S4 to S15: Selfing results in the Aegelsee *Daphnia magna* population: phenotypic and genotypic segregation.

Tables S4 to S12: Selfing results for parents with variation at C and E loci.
Tables S13 and S14: Selfing results for parents with variation at B, C and E loci.
Table S15: Detailed results of statistical analyses applied to F1 groups.

Tables S4 to S12. Expected and observed genotypes and phenotypes of F1 offspring groups resulting from the selfing of F0 parents where variation at the C and E loci was observed (see Supplementary selfing methods Table S21 for clones' details). Observed resistance genotype for parents and offspring was assessed using markers DMPR1 and DMPR3 for the C and the E loci, respectively (see results). Resistance is dominant at the C locus (resistance to C1/C19) whereas resistance is recessive at the E locus (resistance to P20). In addition, an epistatic relationship linking both loci confers susceptibility to P20 to an individual that shows susceptibility to C1 and C19, disregarding the individual genotype at the E locus (see results section Fig. 5). Text in red denotes genotypes and their corresponding phenotypes where the epistatic relationship between the C and the E loci is expected to be revealed in the phenotype. The epistatic relationship "confers" to "cc--" individuals a susceptibility to P20, whatever their genotype at the E locus. Hence the epistasis can only be observed phenotypically in "ccee" offspring. If the epistatic relationship is not present in this case, the observed phenotype would be SS_R, whereas if the epistatic relationship is present, the observed phenotype would be SS_S. **A.** Expected Punnett square for the selfed genotype according to the genetic model. **B.** Expected vs. observed genotypes and phenotypes of selfed F1 offspring. Differences in offspring number between the genotype and the phenotype correspond to instances where either genetic markers analysis did not work, or attachment test was not conducted because the *D. magna* clone got extinct. Expected genotypes and phenotypes were calculated from the Punnett square and using the R package "peas" (Supplementary peas Doc. S1). Genotypes are ordered in upper-case then lower-case fashion. We compared expected vs. observed genotype and resistotype segregation separately in the F1 groups using the Cochran–Mantel–Haenszel (C-M-H) test for repeated tests of independence. When there was only one repeat (Table S7), we used the Fisher test. In cases where there was only one category of expected and observed genotype or phenotype, no test was run (Tables S4, S6, S10, S11 (resistotype only) and S12). In these cases, expectation and observation show a perfect match. Following each C-M-H test, assumption of homogeneity of the odds ratio across repeats was confirmed using a Breslow-Day test (R package DescTools: (Signorell et al. 2018)). However, this test can only be operated in 2x2 tables (phenotype only Tables S5, S7, S8). We then ran a Fisher test of independence for each comparison (expected vs. observed for each repeat, Bonferroni corrected) to detect possible significant differences in opposite directions across repeats, which would result in a non-significant C-M-H test. We did not detect such differences in direction (see Table S15 below for all detailed results of statistical analyses). Tests were run on counts, although we present here segregation of offspring as proportions.

Table S4. Selfed genotype "CCEE". F0 parents: **a**: t3_12.3_1i_12, **b**: t3_12.3_1i_21.

A

RR_S<br>CCEE	CE
CE	RR_S

B

**Markers genotype**	Fraction: Expected	Fraction: Observed, Repeat **a** (n = 43)	Fraction: Observed, Repeat **b** (n = 37)	C-M-H test on counts	**Resistotype**	Fraction: Expected	Fraction: Observed, Repeat **a** (n = 43)	Fraction: Observed, Repeat **b** (n = 37)	C-M-H test on counts
CCEE	1	1	1	NA	RR_S	1	1	1	NA

Table S5. Selfed genotype “CCEe”. F0 parents: **a**: CH-2015-36, **b**: t3_12.3_1, **c**: CH-H-2015-49. Repeat “c” was not tested genotypically as the E locus marker appeared not to be linked to the E locus.

A

RR␣S<br>CCEe	CE	Ce
CE	RR␣S	RR␣S
Ce	RR␣S	RR␣R

B

Markers genotype	Fraction: Expected	Fraction: Observed, Repeat **a** n = 89	**b** n = 31	**c** n = 81	C-M-H test on counts (a+b)	Resistotype	Fraction: Expected	Fraction: Observed, Repeat **a** n = 89	**b** n = 31	**c** n = 79	C-M-H test on counts
CCEE	0.25	0.31	0.19	1	$M^2 = 0.35$, $df = 2$, $p = 0.84$	RR␣S	0.75	0.79	0.74	0.84	$X^2 = 0.85$, $df = 1$, $p = 0.36$
CCEe	0.50	0.46	0.55	0							
CCee	0.25	0.22	0.26	0		RR␣R	0.25	0.21	0.26	0.16	

Table S6. Selfed genotype “CCee”. F0 parents: **a**: CH-H-434-inb2-1, **b**: t1_10.3_2, **c**: CH-H-2015-16, **d**: CH-H-2016-b-70.

A

RR␣R<br>CCee	Ce
Ce	RR␣R

B

Markers genotype	Fraction: Expected	Fraction: Observed, Repeat **a** n = 39	**b** n = 42	**c** n= 66	**d** n = 84	C-M-H test on counts	Resistotype	Fraction: Expected	Fraction: Observed, Repeat **a** n = 39	**b** n = 42	**c** n = 70	**d** n = 79	C-M-H test on counts
CCee	1	1	1	1	1	NA	RR␣R	1	1	1	1	1	NA

Table S7. Selfed genotype “CcEE”. F0 parent: **a**: t2_17.3_4i_12.

A

RR␣S<br>CcEE	CE	cE
CE	RR␣R	RR␣R
cE	RR␣R	SS␣S

B

Markers genotype	Fraction: Expected	Fraction: Observed, Repeat **a** n = 19	Fisher test on counts	Resistotype	Fraction: Expected	Fraction: Observed, Repeat **a** n = 19	Fisher test on counts
CCEE	0.25	0.32	$p = 0.92$	RR␣S	0.75	0.74	$p = 1$
CcEE	0.50	0.42					
ccEE	0.25	0.26		SS␣S	0.25	0.26	

Table S8. Selfed genotype "CcEe". F0 parents: **a**: t3_14.3_1, **b**: t2_17.3_4, **c**: t2_17.3_1, **d**: CH-H-2015-59. Repeat "d" was not tested genotypically as the E locus marker appeared not to be linked to the E locus. Text in grey corresponds to non-matching marker genotypes with their observed resistotypes according to the genetic model. Those non-matching observations are included above in the observed proportions of genotypes.

A

RR␣S CcEe	CE	Ce	cE	ce
CE	RR␣S	RR␣S	RR␣S	RR␣S
Ce	RR␣S	RR␣R	RR␣S	RR␣R
cE	RR␣S	RR␣S	SS␣S	SS␣S
ce	RR␣S	RR␣R	SS␣S	SS␣S

B

Markers genotype	Fraction: Expected	Fraction: Observed, Repeat **a** n = 47	**b** n = 32	**c** n= 80	**d** n = 61	C-M-H test on counts (a+b+c)	Resistotype	Fraction: Expected	Fraction: Observed, Repeat **a** n = 48	**b** n = 34	**c** n = 64	**d** n = 65	C-M-H test on counts
CCEE	0.06	0	0.16	0.05	0.20	$M^2 = 6.79$, $df = 8$, $p = 0.56$	RR␣S	0.56	0.47	0.56	0.65	0.51	$M^2 = 4.61$, $df = 2$, $p = 0.10$
CCEe	0.13	0	0.19	0.12	0								
CcEE	0.13	0.13	0.06	0.18	0.57								
CcEe	0.25	0.40	0.25	0.28	0								
ccEE	0.06	0.09	0	0.03	0.23		SS␣S	0.25	0.30	0.06	0.13	0.21	
ccEe	0.13	0.11	0.03	0.03	0								
ccee	0.06	0.11	0.03	0.03	0								
CCee	0.06	0	0.09	0.17	0		RR␣R	0.19	0.23	0.38	0.22	0.28	
Ccee	0.13	0.17	0.19	0.10	0								
CCEE					0.11		RR␣R						
CCEe			0.06				RR␣R						
CcEE		0.02	0.03		0.16		RR␣R						
CcEe		0.06	0.06				RR␣R						
CCee				0.05			RR␣S						
Ccee		0.02	0.06				RR␣S						
CcEE				0.02	0.02		SS␣S						
CcEe				0.02			SS␣S						
ccEE					0.03		RR␣S						

Table S9. Selfed genotype "Ccee". F0 parents: **a**: t1_10.3_4, **b**: t5_10.3_3, **c**: CH-H-434-inb2-2.

A

RR␣R Ccee	Ce	ce
Ce	RR␣R	RR␣R
ce	RR␣R	SS␣S

B

Markers genotype	Fraction: Expected	Fraction: Observed, Repeat **a** n = 36	**b** n = 49	**c** n= 21	C-M-H test on counts	Resistotype	Fraction: Expected	Fraction: Observed, Repeat **a** n = 36	**b** n = 49	**c** n= 22	C-M-H test on counts
CCee	0.25	0.19	0.16	0.14	$M^2 = 2.16$, $df = 2$, $p = 0.34$	RR␣R	0.75	0.75	0.80	0.62	$X^2 = 0.0062$, $df = 1$, $p = 0.94$
Ccee	0.50	0.56	0.63	0.48							
ccee	0.25	0.25	0.20	0.38		SS␣S	0.25	0.25	0.20	0.38	

Table S10. Selfed genotype “ccEE”. F0 parent: **a**: CH-H-2015-97.

A

SS␣S ccEE	cE
cE	SS␣S

B

Markers genotype	Fraction: Expected	Fraction: Observed, Repeat **a** n = 89	Fisher test on counts	Resistotype	Fraction: Expected	Fraction: Observed, Repeat **a** n = 87	Fisher test on counts
ccEE	1	1	NA	SS␣S	1	1	NA

Table S11. Selfed genotype “ccEe”. F0 parents: **a**: CH-H-2015-113, **b**: t4_10.3_16.

A

SS␣S ccEe	cE	ce
cE	SS␣S	SS␣S
ce	SS␣S	SS␣S

B

Markers genotype	Fraction: Expected	Fraction: Observed, Repeat **a** n = 82	Fraction: Observed, Repeat **b** n = 63	C-M-H test on counts	Resistotype	Fraction: Expected	Fraction: Observed, Repeat **a** n = 84	Fraction: Observed, Repeat **b** n = 65	C-M-H test on counts
ccEE	0.25	0.27	0.15	$M^2 = 0.58$, $df = 2$, $p = 0.75$	SS␣S	1	1	1	NA
ccEe	0.50	0.55	0.44						
ccee	0.25	0.18	0.41						

Table S12. Selfed genotype “ccee”. F0 parents: **a**: t4_13.3_2, **b**: CH-H-2015-86.

A

SS␣S ccee	ce
ce	SS␣S

B

Markers genotype	Fraction: Expected	Fraction: Observed, Repeat **a** n = 76	Fraction: Observed, Repeat **b** n = 36	C-M-H test on counts	Resistotype	Fraction: Expected	Fraction: Observed, Repeat **a** n = 74	Fraction: Observed, Repeat **b** n = 35	C-M-H test on counts
ccee	1	1	1	NA	SS␣S	1	1	1	NA

Tables S13 and S14. Expected and observed phenotypes of F1 offspring resulting from the selfing of F0 parents where variation at the B- and E loci was observed (see Supplementary selfing methods Table S21 for clones' details). Individuals bearing the dominant allele at the B locus display a SR⎵⎵ resistotype underlined by a "--B-cc" genotype. In the Swisspond population we assume the recessive allele is fixed at the A locus and the dominant allele at the B locus is rare (see results section). Individuals with a SR⎵⎵ resistotype are thus expected to possess an "aaB-cc" genotype in our study population. As genetic markers were not designed to discriminate variation at the B locus and consequently do not show linkage with expected genotypes, we present only phenotype segregation. We deduced the resistance genotypes of the present F0 parents based on their resistotype and on the segregation patterns of their F1 offspring. In these F1 offspring, we observed SR⎵R individuals, supposedly "aaB-ccee", which indicates that the epistatic relationship described before between the C and the E loci (results section Fig. 5) should also include the B locus. If the epistasis was only between the C- and the E locus, SR⎵⎵ individuals would necessarily be SR⎵S. However, we do find SR⎵R individuals among F1 offspring of SR⎵S parents, which indicates that the "bbcc--" genotype induces susceptibility to P20, regardless of the genotype at the E locus. As SR⎵⎵ individuals are rare in the population, we base this hypothesis solely on the selfed offspring presented below. As the recessive allele at the A locus is fixed in the population, we cannot infer a possible role of the A locus in this epistatic relationship. Text in red denotes phenotypes and genotypes where this epistatic relationship between the B/C and the E loci is expected to be revealed in the phenotype. The epistatic relationship "confers" to "bbcc--" individuals a susceptibility to P20, whatever their genotype at the E locus. Hence the epistasis can only be observed phenotypically in "bbccee" offspring. If the epistatic relationship was not present in this case, the observed phenotype would be SS⎵R, whereas if the epistatic relationship is present, the observed phenotype is SS⎵S. This genetic model is presented in Fig. 5 in the results section. **A.** Expected Punnett square for the selfed genotype according to the genetic model including the B locus. **B.** Expected and observed phenotypes of selfed F1 offspring. Expected phenotypes were calculated from the Punnett square and using the R package "peas" (Supplementary peas Doc. S2). Genotypes are ordered in upper-case then lower-case fashion. We compared expected vs. observed resistotype segregation in the F1 groups using the Fisher test of independence (see Table S15 below for all detailed results of statistical analyses). Tests were run on counts, although we present here segregation of offspring as proportions.

Table S13. Selfed genotype "BbccEE". F0 parent: **a**: t0_9.3_7.

A

SR⎵S BbccEE	BcE	bcE
BcE	SR⎵S	SR⎵S
bcE	SR⎵S	SS⎵S

B

Markers genotype	Fraction: Expected	Fraction: Observed, Repeat, a, n = 35	Fisher test on counts	Resistotype	Fraction: Expected	Fraction: Observed, Repeat, a, n = 37	Fisher test on counts
BBccEE	0.25	NA		SR⎵S	0.75	0.76	
BbccEE	0.50	NA	NA	SS⎵S	0.25	0.22	$p = 1$
bbccEE	0.25	NA		SR⎵R	0.00	0.03	

Table S14. Selfed genotype "BbccEe". F0 parent: **a**: t0_28.2_43.

A

SR_S BbccEe	BcE	Bce	bcE	bce
BcE	SR_S	SR_S	SR_S	SR_S
Bce	SR_S	SR_R	SR_S	SR_R
bcE	SR_S	SR_S	SS_S	SS_S
bce	SR_S	SR_R	SS_S	SS_S

B

Markers genotype	Fraction: Expected	Fraction: Observed, Repeat **a** n = 36	Fisher test on counts	Resistotype	Fraction: Expected	Fraction: Observed, Repeat **a** n = 38	Fisher test on counts
BBccEE	0.06	NA		SR_S	0.56	0.55	
BBccEe	0.13	NA					
BbccEE	0.13	NA					
BbccEe	0.25	NA					
bbccEE	0.06	NA	NA	SS_S	0.25	0.32	p = 0.58
bbccEe	0.13	NA					
bbccee	0.06	NA					
BBccee	0.06	NA		SR_R	0.19	0.13	
Bbccee	0.13	NA					

Table S15. Detailed results of statistical analyses applied to F1 offspring groups. Analyses are described in tables' legends above.

Phenotype

	CMH test				BD test			Fisher test: repeat a		repeat b		repeat c		repeat d	
	M2	*df*	*p*		*X²*	*df*	*p*	*p*	*p + Bonf.*	*p*	*p + Bonf.*	*p*	*p + Bonf.*	*p*	*p + Bonf.*
Table S4	NA	NA	NA	NA	NA	NA	NA	NA	NA	NA	NA	NA	NA	NA	NA
Table S5	X^2 = 0.85	1	0.36	CI = (0.49, 1.25), estimate = 0.78, null value = 1	1	2	1	1	1	1	1	0	0.72	NA	NA
Table S6	NA	NA	NA	NA	NA	NA	NA	NA	NA	NA	NA	NA	NA	NA	NA
Table S7	NA	NA	NA	NA	NA	NA	NA	1	NA	NA	NA	NA	NA	NA	NA
Table S8	4.61	2	0.1	NA	NA	NA	NA	1	1	0	0.27	0	1	0	1
Table S9	X^2 = 0.0062	1	0.94	CI = (0.55, 1.9), estimate = 1.03, null value = 1	1	2	1	1	1	1	1	1	1	NA	NA
Table S10	NA	NA	NA	NA	NA	NA	NA	NA	NA	NA	NA	NA	NA	NA	NA
Table S11	NA	NA	NA	NA	NA	NA	NA	NA	NA	NA	NA	NA	NA	NA	NA
Table S12	NA	NA	NA	NA	NA	NA	NA	NA	NA	NA	NA	NA	NA	NA	NA
Table S13	NA	NA	NA	NA	NA	NA	NA	1	NA	NA	NA	NA	NA	NA	NA
Table S14	NA	NA	NA	NA	NA	NA	NA	1	NA	NA	NA	NA	NA	NA	NA

Genotype

	CMH test			Fisher test: repeat a		repeat b		repeat c	
	M²	*df*	*p*	*p*	*p + Bonf.*	*p*	*p + Bonf.*	*p*	*p + Bonf.*
Table S4	NA	NA	NA	NA	NA	NA	NA	NA	NA
Table S5	0.35	2	0.84	1	1	1	1	NA	NA
Table S6	NA	NA	NA	NA	NA	NA	NA	NA	NA
Table S7	NA	NA	NA	1	NA	NA	NA	NA	NA
Table S8	6.79	8	0.56	0	0.19	1	1	0	1
Table S9	2.16	2	0.34	1	1	0	1	1	1
Table S10	NA	NA	NA	NA	NA	NA	NA	NA	NA
Table S11	0.58	2	0.75	1	1	0	0.25	NA	NA
Table S12	NA	NA	NA	NA	NA	NA	NA	NA	NA
Table S13	NA	NA	NA	NA	NA	NA	NA	NA	NA
Table S14	NA	NA	NA	NA	NA	NA	NA	NA	NA

Linking the genomic regions and the genetic model of resistance

Table S16. Multi-marker genotypes and their expected corresponding resistance genotype at the C and E loci and resistotypes (resistance phenotypes) of *Daphnia magna* in the Swisspond population, assuming perfect linkage between markers and resistance loci. All possible allele combinations for the four markers DMPR1, 2, 3 and 4 (DMPR: *Daphnia magna* - *Pasteuria ramosa*). DMPR1 and 2 are physically linked to the C locus while DMPR3 and 4 are physically linked to the E locus. Zeros (0) and ones (1) correspond to absence and presence of an allele, respectively. The size of an amplicon containing an allele is denoted next to the name of the allele (see Supplementary GWAS methods for details on the markers). For example, DMPR1_R1_89 corresponds to the amplicon of 89 bp yielded by the PCR for the first R allele of the DMPR1 marker. Inferred resistotypes in red denote resistotypes where the epistatic interaction between the C and the E loci is detectable. In order to show the logic of the different possible combinations, lines with zeros and ones identical to the line directly above them are shown in grey.

Alleles associated to the C locus					Alleles associated to the E locus							
DMPR1_R1_89	DMPR1_R2_113	DMPR1_S_118	DMPR2_S_176	DMPR2_R_184	DMPR3_S_206	DMPR3_R1_211	DMPR3_R2_212	DMPR4_R_128	DMPR4_S_136	Multi-marker genotype	Inferred resistance genotype	Inferred resistotype
**0**	**0**	**1**	**1**	**0**	**0**	**0**	**1**	**1**	**0**	001_10_001_10	ccee	SS_S
0	0	1	1	0	**0**	**1**	**0**	1	0	001_10_010_10	ccee	SS_S
0	0	1	1	0	**0**	**1**	**1**	1	0	001_10_011_10	ccee	SS_S
0	0	1	1	0	**1**	**0**	**0**	**0**	**1**	001_10_100_01	ccEE	SS_S
0	0	1	1	0	**1**	**0**	**1**	**1**	**1**	001_10_101_11	ccEe	SS_S
0	0	1	1	0	**1**	**1**	**0**	1	1	001_10_110_11	ccEe	SS_S
**0**	**1**	**0**	**0**	**1**	**0**	**0**	**1**	**1**	**0**	010_01_001_10	CCee	RR_R
0	1	0	0	1	**0**	**1**	**0**	1	0	010_01_010_10	CCee	RR_R
0	1	0	0	1	**0**	**1**	**1**	1	0	010_01_011_10	CCee	RR_R
0	1	0	0	1	**1**	**0**	**0**	**0**	**1**	010_01_100_01	CCEE	RR_S
0	1	0	0	1	**1**	**0**	**1**	**1**	**1**	010_01_101_11	CCEe	RR_S
0	1	0	0	1	**1**	**1**	**0**	1	1	010_01_110_11	CCEe	RR_S
**0**	**1**	**1**	**1**	**1**	**0**	**0**	**1**	**1**	**0**	011_11_001_10	Ccee	RR_R
0	1	1	1	1	**0**	**1**	**0**	1	0	011_11_010_10	Ccee	RR_R
0	1	1	1	1	**0**	**1**	**1**	1	0	011_11_011_10	Ccee	RR_R
0	1	1	1	1	**1**	**0**	**0**	**0**	**1**	011_11_100_01	CcEE	RR_S
0	1	1	1	1	**1**	**0**	**1**	**1**	**1**	011_11_101_11	CcEe	RR_S
0	1	1	1	1	**1**	**1**	**0**	1	1	011_11_110_11	CcEe	RR_S
**1**	**0**	**0**	**0**	**1**	**0**	**0**	**1**	**1**	**0**	100_01_001_10	CCee	RR_R
1	0	0	0	1	**0**	**1**	**0**	1	0	100_01_010_10	CCee	RR_R
1	0	0	0	1	**0**	**1**	**1**	1	0	100_01_011_10	CCee	RR_R
1	0	0	0	1	**1**	**0**	**0**	**0**	**1**	100_01_100_01	CCEE	RR_S
1	0	0	0	1	**1**	**0**	**1**	**1**	**1**	100_01_101_11	CCEe	RR_S
1	0	0	0	1	**1**	**1**	**0**	1	1	100_01_110_11	CCEe	RR_S
**1**	**0**	**1**	**1**	**1**	**0**	**0**	**1**	**1**	**0**	101_11_001_10	Ccee	RR_R
1	0	1	1	1	**0**	**1**	**0**	1	0	101_11_010_10	Ccee	RR_R
1	0	1	1	1	**0**	**1**	**1**	1	0	101_11_011_10	Ccee	RR_R
1	0	1	1	1	**1**	**0**	**0**	**0**	**1**	101_11_100_01	CcEE	RR_S
1	0	1	1	1	**1**	**0**	**1**	**1**	**1**	101_11_101_11	CcEe	RR_S
1	0	1	1	1	**1**	**1**	**0**	1	1	101_11_110_11	CcEe	RR_S
**1**	**1**	**0**	**0**	**1**	**0**	**0**	**1**	**1**	**0**	110_01_001_10	CCee	RR_R
1	1	0	0	1	**0**	**1**	**0**	1	0	110_01_010_10	CCee	RR_R
1	1	0	0	1	**0**	**1**	**1**	1	0	110_01_011_10	CCee	RR_R
1	1	0	0	1	**1**	**0**	**0**	**0**	**1**	110_01_100_01	CCEE	RR_S
1	1	0	0	1	**1**	**0**	**1**	**1**	**1**	110_01_101_11	CCEe	RR_S
1	1	0	0	1	**1**	**1**	**0**	1	1	110_01_110_11	CCEe	RR_S

Table S17. Observed allele combinations for markers DMPR1 and 2 and their corresponding inferred resistance genotype, expected and observed resistotypes. DMPR1 and 2 are linked to the C locus. Details about the markers and their expected signatures from the genetic model of resistance are given in Supplementary GWAS methods. Text in grey represents occurrences where the measured and expected resistotypes do not match. For each genotype, the expected count of resistotypes is calculated as the total count of observed resistotypes. The panel of clones used represents 24 selfed offspring groups (F1) ranging from 19 to 89 individual clones (Supplementary selfing methods), as well as three random samples from the Swisspond from 2014 to 2016 (22 to 108 clones), either hatched from resting eggs of directly sampled from the pond. Differences in total sample size correspond to occurrences where a marker did not amplify or when the marker signature could not be clearly identified. The initial total sample size of phenotyped clones is n = 1550. Individuals with a “SR_ _” resistotype were excluded from the analysis as they were not used in the GWAS from which the markers were designed. Better linkage is observed between the C locus and DMPR1 than between the C locus and DMPR2.

DMPR1_R1_89	DMPR1_R2_113	DMPR1_S_118	DMPR1 genotype	Inferred resistance genotype	Inferred resistotype	Measured resistotype	Count (n = 1442)	Expected count from inferred resistotypes	Frequency (n = 1442)	Expected frequency from inferred resistotypes
**0**	**0**	**1**	001	cc	SS_ _	SS_ _	488	490	0.338	0.340
						RR_ _	*2*	*0*	*0.001*	*0*
**0**	**1**	**0**	010	CC	RR_ _	RR_ _	515	515	0.357	0.357
**0**	**1**	**1**	011	Cc	RR_ _	RR_ _	251	254	0.174	0.176
						SS_ _	*3*	*0*	*0.002*	*0*
**1**	**0**	**0**	100	CC	RR_ _	RR_ _	76	76	0.053	0.053
**1**	**0**	**1**	101	Cc	RR_ _	RR_ _	67	68	0.046	0.047
						SS_ _	*1*	*0*	*0.001*	*0*
**1**	**1**	**0**	110	CC	RR_ _	RR_ _	39	39	0.027	0.027
							Fisher’s exact test $p = 0.77$			

DMPR2_S_176	DMPR2_R_184		DMPR2 genotype	Inferred resistance genotype	Inferred resistotype	Measured resistotype	Count (n = 1434)	Expected count from inferred resistotypes	Frequency (n = 1434)	Expected frequency from inferred resistotypes
**0**	**1**		01	CC	RR_ _	RR_ _	548	549	0.382	0.383
						SS_ _	*1*	*0*	*0.001*	*0*
**1**	**0**		10	cc	SS_ _	SS_ _	480	599	0.335	0.418
						RR_ _	*119*	*0*	*0.083*	*0*
**1**	**1**		11	Cc	RR_ _	RR_ _	283	286	0.197	0.199
						SS_ _	*3*	*0*	*0.002*	*0*
							Fisher’s exact test $p = 1.25.10^{-37}$			

Table S18. Observed allele combinations for markers DMPR3 and 4 and their corresponding inferred resistance genotype, expected and observed resistotypes. DMPR1 and 2 are linked to the E locus. Details about the markers and their expected signatures from the genetic model of resistance are given in Supplementary GWAS methods. Within the groups of "ee" genotypes, we separately present the "SS__" C1/C19 resistotype as we expect in that case the P20 resistotype to be "___S", due to the epistatic relationship between the C and the E locus. Text in grey represents occurrences where the measured and expected resistotypes do not match. Text in red denotes resistotypes where the epistatic interaction between the C and the E loci is detectable. For each genotype, the expected count of resistotypes is calculated as the total count of observed resistotypes. The panel of clones used represents 24 selfed offspring groups (F1) ranging from 12 to 84 individual clones (Supplementary selfing methods), as well as three random samples from the Swisspond from 2014 to 2016 (22 to 108 clones), either hatched from resting eggs of directly sampled in the pond. Differences in total sample size correspond to occurrences where a marker did not amplify or when the marker signature could not be clearly identified. The initial total sample size of phenotyped clones is n = 1550. Individuals with a "SR__" resistotype were excluded from the analysis as they were not used in the GWAS from which the markers were designed. Better linkage is observed between the E locus and DMPR3 than between the E locus and DMPR4. Note that marker DMPR4 did not amplify as well as the other markers (n = 911). This marker showed better amplification when put alone in a PCR reaction however we decided not to use it further as it showed poor linkage to the E locus.

**DMPR3_S_206**	**DMPR3_R1_211**	**DMPR3_R2_212**	**DMPR3 genotype**	**Inferred resistance genotype**	**Measured resistotype to C1/C19**	**Inferred resistotype to P20**	**Measured resistotype to P20**	**Count (n = 1438)**	**Expected count from inferred resistotypes**	**Frequency (n = 1438)**	**Expected frequency from inferred resistotypes**
**0**	**1**	**0**	010	ee	RR__	___R	___R	468	481	0.325	0.334
							___S	*13*	*0*	*0.009*	*0*
					SS__	___S	___S	227	227	0.158	0.158
							___R				
**0**	**1**	**1**	011	ee	RR__	___R	___R	3	3	0.002	0.002
**1**	**0**	**0**	100	EE	XX__	___S	___S	417	450	0.290	0.313
							___R	*33*	*0*	*0.023*	*0*
**1**	**0**	**1**	101	Ee	XX__	___S	___S	1	1	0.001	0.001
**1**	**1**	**0**	110	Ee	XX__	___S	___S	247	276	0.172	0.192
							___R	*29*	*0*	*0.020*	*0*
								Fisher's exact test $p = 5.34.10^{-19}$			

**DMPR4_R_128**	**DMPR4_S_136**	**DMPR4 genotype**	**Inferred resistance genotype**	**Measured resistotype to C1/C19**	**Inferred resistotype to P20**	**Measured resistotype to P20**	**Count (n = 911)**	**Expected count from inferred resistotypes**	**Frequency (n = 911)**	**Expected frequency from inferred resistotypes**
**0**	**1**	01	EE	XX__	___S	___S	357	374	0.392	0.411
						___R	*17*	*0*	*0.019*	*0*
**1**	**0**	10	ee	RR__	___R	___R	18	19	0.020	0.021
						___S	*1*	*0*	*0.001*	*0*
				SS__	___S	___S	6	6	0.007	0.007
**1**	**1**	11	Ee	XX__	___S	___S	251	512	0.276	0.562
						___R	*261*	*0*	*0.286*	*0*
							Fisher's exact test $p = 1.00.10^{-100}$			

Methods

Assessment of resistotype segregation

Table S19. Overview of the parent clones used to produce the selfed offspring sorted by their inferred genotypes. Resistotype corresponds to the resistance phenotype to the *Pasteuria ramosa* isolates C1, C19 and P20, in that order. The inferred genotypes at the C and E loci are the result of the markers analysis of the parent clones (see Supplementary markers Tables S22 to S25). Two exceptions (marked with a "*") are cases where the marker genotype at the E locus was "EE", although segregation in the selfed F1 offspring revealed a "Ee" genotype. We selfed one to four parents of each homozygous genotype category, and one to four parents of each simple or double heterozygous genotype category. The genotype of the two "SR_S" parents was inferred using only the segregation pattern of their selfed offspring. The markers were designed to map resistance to C1, C19 and P20 in the Aegelsee population, using the three main resistotypes only: RR_S, RR_R and SS_S, and were consequently not linked to the genotype in SR_ _ individuals. F0 parents correspond to females caught from plankton (clone name starting with "CH") or hatched from resting eggs (clone name starting with "t") collected from the natural population between 2011 and 2016. Differences in offspring number between the genotype and the phenotype correspond to instances where either genetic markers analysis did not work, or attachment test was not conducted because the *D. magna* clone got extinct.

Resistotype	Inferred genotype	Repeat name	Parent clone name	n (offspring) Genotype n = 1274	n (offspring) Phenotype n = 1266	
RR_S	CCEE	a	t3_12.3_1i_12	43	43	
		b	t3_12.3_1i_21	37	37	
RR_S	CCEe	a	CH-2015-36	89	89	
		b	t3_12.3_1	31	31	
		c	CH-H-2015-49	81	79	*
RR_R	CCee	a	CH-H-434-inb2-1	39	39	
		b	t1_10.3_2	42	42	
		c	CH-H-2015-16	66	70	
		d	CH-H-2016-b-70	84	79	
RR_S	CcEE	a	t2_17.3_4i_12	19	19	
RR_S	CcEe	a	t3_14.3_1	47	48	
		b	t2_17.3_4	32	34	
		c	t2_17.3_1	80	64	
		d	CH-H-2015-59	61	65	*
RR_R	Ccee	a	t1_10.3_4	36	36	
		b	t5_10.3_3	49	49	
		c	CH-H-434-inb2-2	21	22	
SS_S	ccEE	a	CH-H-2015-97	89	87	
SS_S	ccEe	a	CH-H-2015-113	82	84	
		b	t4_10.3_16	63	65	
SS_S	ccee	a	t4_13.3_2	76	74	
		b	CH-H-2015-86	36	35	
SR_S	BbccEE	a	t0_9.3_7	35	37	
SR_S	BbccEe	a	t0_28.2_43	36	38	

Documents S1 and S2: Implementation of the genetic model of resistance using the “peas” package.

Document S1: Variation at the C and E loci
Document S2: Variation at the B, C and E loci

Document S1. Implementation of the genetic model of resistance using the “peas” package. Variation at the C and E loci. The R package “peas” is available at https://github.com/JanEngelstaedter/peas. We consider variation at the C and E loci. In **step 2.1** we define the genetic system as two loci: C and E presenting each two alleles. Both loci sit on different linkage groups. In **step 2.2** we set the genetic model itself, defining resistance genotypes and their corresponding resistance phenotypes (resistotypes). Resistotypes are presented as resistant (R) or susceptible (S) to the bacteria strains C1, C19 and P20, in that order. Resistance to the bacteria is dominant for the C locus (Metzger et al. 2016) whereas resistance is recessive at the E locus. The dominant allele at the C locus confers resistance to C1 and C19. The recessive allele at the E locus confers resistance to P20. The epistatic interaction acts between the C and the E loci. Recessive individuals at C locus are susceptible to P20, regardless of their genotype at the E locus. The genetic model produces 9 possible genotypes coding for 3 different resistotypes. The number of possible genotypes corresponds to the draw of one element among three (AA, Aa or aa genotype) two consecutive times (at two different loci): $n(possible\ genotypes) = 2\binom{3}{1} = 9$. In **step 3** we produce the expected results from the selfing of F0 mothers with all possible combinations of the two loci C and E and store them in a spreadsheet (Results section Table 1 & Supplementary selfing results: Tables S4 to S12).

Genetic model for resistance to P20 in the Aegelsee

implemented in peas R package

1. Install the package

```
#install.packages("devtools")
#devtools::install_github("JanEngelstaedter/peas", build_vignettes = TRUE)

library(peas)
```

C_E genetic model of resistance

2. Set up the genetic model

2.1 Defining the genetic system

```
# 2 loci with 2 alleles each, on different chromosomes
CE <- newGenopheno(nloci = 1,
            alleleNames = list(c("c", "C")))
CE <- addLinkageGroup(CE, alleleNames = list(c("e", "E")))
CE
## Genetic system comprising 2 linkage groups:
##   Linkage group 1: autosomal, 1 locus
##     Alleles at locus 1: c, C
##   Linkage group 2: autosomal, 1 locus
##     Alleles at locus 1: e, E
## No phenotypes defined.
```

2.2 Set genotypes and their corresponding phenotypes: THE GENETIC MODEL

```
CE <- setPhenotypes(CE, "S/R", "__|__","SS_R") # default all recessive --> SS_R
# (we don't take the epistasis relation into account, this will be the last line of the model)
```

```
CE <- setPhenotypes(CE, "S/R", "C_ | __","RR_R") # C --> R to C1 and C19

CE <- setPhenotypes(CE, "S/R", "C_ | E_","RR_S") # E --> S to P20


CE <- setPhenotypes(CE, "S/R", "cc | __","SS_S") # cc --> S to P20, hides E
```

Summary of model

```
CE
## Genetic system comprising 2 linkage groups:
##   Linkage group 1: autosomal, 1 locus
##     Alleles at locus 1: c, C
##   Linkage group 2: autosomal, 1 locus
##     Alleles at locus 1: e, E
## Phenotypes defined for the following traits:
##   S/R (trait values: SS_S, RR_R, RR_S)
```

List of all possible genotype combinations and corresponding phenotypes

```
CEallgeno<-getPhenotypes(CE)
CEallgeno # all possible combinations
##         S/R
## cc | ee SS_S
## Cc | ee RR_R
## CC | ee RR_R
## cc | Ee SS_S
## Cc | Ee RR_S
## CC | Ee RR_S
## cc | EE SS_S
## Cc | EE RR_S
## CC | EE RR_S
nrow(CEallgeno) # 9 possible genotypes
## [1] 9
nrow(unique(CEallgeno)) # 3 possible resistotypes
## [1] 3
```

3. Predict crosses

```
CEcombi<-row.names(CEallgeno) # all possible geno combinations from the model
CEcombi<-c(CEcombi[9],CEcombi[6],CEcombi[3],
        CEcombi[8],CEcombi[5],CEcombi[2],
        CEcombi[7],CEcombi[4],CEcombi[1]) # reorder them (optional)
allcrossCE<-list() # create empty list to  store the crossing results

library(xlsx)
# for all the nine possible combinations with C and E varying (F0 mothers)
# we calculate the genotypes and phenotypes fractions in the F1 offspring with the predictCross
function (from the selfing of F0 mothers)

for (i in 1:9) {
# using the "CE" genetic model, we cross the "i" combination with itself
  allcrossCE[[i]]<-predictCross(CE, CEcombi[i], CEcombi[i])

# we add the corresponding resistotypes to the genotype output
  allcrossCE[[i]]$genotypes$SRtrait <- getPhenotypes(CE, equivalent =
"none")[row.names(allcrossCE[[i]]$genotypes), ]
```

```r
# we store the result in a spreadsheet (genotype output)
 write.xlsx(allcrossCE[[i]]$genotypes,"CEcross.xlsx", sheetName= paste(CEcombi[i],"geno",
as.character(i)), append=T)

# then the phenotype output in another sheet of the same document
 write.xlsx(allcrossCE[[i]]$phenotypes,"CEcross.xlsx", sheetName= paste(CEcombi[i],"pheno",
as.character(i)), append=T)
 }
```

```
allcrossCE
## [[1]]
## [[1]]$genotypes
##         fraction SRtrait
## CC | EE        1    RR_S
##
## [[1]]$phenotypes
##    S/R fraction
## 1 RR_S        1
##
##
## [[2]]
## [[2]]$genotypes
##         fraction SRtrait
## CC | EE     0.25    RR_S
## CC | Ee     0.50    RR_S
## CC | ee     0.25    RR_R
##
## [[2]]$phenotypes
##    S/R fraction
## 1 RR_R     0.25
## 2 RR_S     0.75
##
##
## [[3]]
## [[3]]$genotypes
##         fraction SRtrait
## CC | ee        1    RR_R
##
## [[3]]$phenotypes
##    S/R fraction
## 1 RR_R        1
##
##
## [[4]]
## [[4]]$genotypes
##         fraction SRtrait
## CC | EE     0.25    RR_S
## Cc | EE     0.50    RR_S
## cc | EE     0.25    SS_S
##
## [[4]]$phenotypes
##    S/R fraction
## 1 RR_S     0.75
## 2 SS_S     0.25
##
##
## [[5]]
## [[5]]$genotypes
##         fraction SRtrait
## CC | EE   0.0625    RR_S
## Cc | EE   0.1250    RR_S
## CC | Ee   0.1250    RR_S
## Cc | Ee   0.2500    RR_S
## cc | EE   0.0625    SS_S
## cc | Ee   0.1250    SS_S
## CC | ee   0.0625    RR_R
## Cc | ee   0.1250    RR_R
## cc | ee   0.0625    SS_S
##
## [[5]]$phenotypes
##    S/R fraction
## 1 RR_R   0.1875
## 2 RR_S   0.5625
## 3 SS_S   0.2500
##
##
## [[6]]
## [[6]]$genotypes
##         fraction SRtrait
## CC | ee     0.25    RR_R
## Cc | ee     0.50    RR_R
## cc | ee     0.25    SS_S
##
## [[6]]$phenotypes
##    S/R fraction
## 1 RR_R     0.75
## 2 SS_S     0.25
##
##
## [[7]]
## [[7]]$genotypes
##         fraction SRtrait
## cc | EE        1    SS_S
##
## [[7]]$phenotypes
##    S/R fraction
## 1 SS_S        1
##
##
## [[8]]
## [[8]]$genotypes
##         fraction SRtrait
## cc | EE     0.25    SS_S
## cc | Ee     0.50    SS_S
## cc | ee     0.25    SS_S
##
## [[8]]$phenotypes
##    S/R fraction
## 1 SS_S        1
##
##
## [[9]]
## [[9]]$genotypes
##         fraction SRtrait
## cc | ee        1    SS_S
##
## [[9]]$phenotypes
##    S/R fraction
## 1 SS_S        1
```

BC_E genetic model of resistance

Document S2. Implementation of the genetic model of resistance using the "peas" package. Variation at the B, C and E loci. In **step 2.1** we define the genetic system as three loci: B, C and E presenting each two alleles. The B and C loci sit on the same linkage group and recombination rate between them is set to r2 = (1-exp(-2*23.1/100))/2 as calculated in (Metzger et al. 2016). The E locus sits on a different linkage group. In **step 2.2** we set the genetic model itself, defining resistance genotypes and their corresponding resistance phenotypes (resistotypes). Resistotypes are presented as resistant (R) or susceptible (S) to the bacteria strains C1, C19 and P20, in that order. Resistance to the bacteria is dominant for the B and C loci (Metzger et al. 2016) whereas resistance is recessive at the E locus. The dominant allele at the B locus confers resistance to C19. The first epistatic interaction acts between the B and C loci. The dominant allele at the C locus confers resistance to C1 and C19, regardless of the genotype at the B locus. The recessive allele at the E locus confers resistance to P20. The second epistatic interaction acts between the B/C loci and the E locus. Double recessive individuals at the B and C loci are susceptible to P20, regardless of their genotype at the E locus. The genetic model produces 27 possible genotypes coding for 5 different resistotypes. The number of possible genotypes corresponds to the draw of one element among three (AA, Aa or aa genotype) three consecutive times (at three different loci): $n(possible\ genotypes) = 3\binom{3}{1} = 27$. In **step 3** we produce the expected results from the selfing of F0 mothers with all possible combinations of the three loci B, C and E and store them in a spreadsheet. From these we extract the results of the F1 selfed offspring groups produced in the present study (Results section Table 2 & Supplementary selfing results Table S13 and S14).

2. Set up the genetic model

2.1 Defining the genetic system

```
library(peas)
r2<-(1-exp(-2*23.1/100))/2 ## recombination rate between B and C loci (Metzger et al. 2016)

# 3 loci with 2 alleles each, BC clustered together (Metzger et al. 2016).
BCE <- newGenopheno(nloci = 2,
            alleleNames = list(c("b", "B"), c("c", "C")),
            rec = r2)
BCE <- addLinkageGroup(BCE, alleleNames = list(c("e", "E")))
```

2.2 Set genotypes and their corresponding phenotypes: THE GENETIC MODEL

```
BCE <- setPhenotypes(BCE, "S/R", "__~__|__","SS_R") # default all recessive --> SS_R
# (we don't take the epistasis relation into account, this will be the last line of the model)

BCE <- setPhenotypes(BCE, "S/R", "B_~__|__","SR_R") # B --> R to C19

BCE <- setPhenotypes(BCE, "S/R", "B_~__|E_","SR_S") # E --> S to P20

BCE <- setPhenotypes(BCE, "S/R", "__~C_|__","RR_R") # C hides B and --> R to C1 and C19

BCE <- setPhenotypes(BCE, "S/R", "__~C_|E_","RR_S") # E --> S to P20

BCE <- setPhenotypes(BCE, "S/R", "bb~cc|__","SS_S") # bb and cc --> S to P20, hides E
```

Summary of model

```
BCE
## Genetic system comprising 2 linkage groups:
##   Linkage group 1: autosomal, 2 loci with recombination rate 0.1849888
##     Alleles at locus 1: b, B
##     Alleles at locus 2: c, C
##   Linkage group 2: autosomal, 1 locus
##     Alleles at locus 1: e, E
## Phenotypes defined for the following traits:
##   S/R (trait values: SS_S, SR_R, RR_R, SR_S, RR_S)
```

List of all possible genotype combinations and corresponding phenotypes

```
BCEallgeno<-getPhenotypes(BCE)
nrow(BCEallgeno) # 27 possible genotypes
## [1] 27
nrow(unique(BCEallgeno)) # 5 possible resistotypes
## [1] 5
```

3. Predict crosses

```
BCEcombi<-row.names(BCEallgeno) # all possible geno combinations from the model
allcrossBCE<-list() # create empty list to  store the crossing results

library(xlsx)

for (i in 1:27) {
# using the "BCE" genetic model, we cross the "i" combination with itself
  allcrossBCE[[i]]<-predictCross(BCE, BCEcombi[i], BCEcombi[i])

# we add the corresponding resistotypes to the genotype output
  allcrossBCE[[i]]$genotypes$SRtrait <- getPhenotypes(BCE, equivalent =
"none")[row.names(allcrossBCE[[i]]$genotypes), ]

# we store the result in a spreadsheet (genotype output)
  write.xlsx(allcrossBCE[[i]]$genotypes,"BCEcross.xlsx", sheetName=
paste(BCEcombi[i],"geno", as.character(i)), append=T)

# then the phenotype output in another sheet of the same document
  write.xlsx(allcrossBCE[[i]]$phenotypes,"BCEcross.xlsx", sheetName=
paste(BCEcombi[i],"pheno", as.character(i)), append=T)
}
```

```
# get a specific crossing

match("Bb~cc | Ee", BCEcombi) # position
11
## [1] 11
allcrossBCE[[11]]
## $genotypes
##          fraction SRtrait
## BB~cc | EE   0.0625    SR_S
## Bb~cc | EE   0.1250    SR_S
## BB~cc | Ee   0.1250    SR_S
## Bb~cc | Ee   0.2500    SR_S
## bb~cc | EE   0.0625    SS_S
## bb~cc | Ee   0.1250    SS_S
## BB~cc | ee   0.0625    SR_R
## Bb~cc | ee   0.1250    SR_R
## bb~cc | ee   0.0625    SS_S
##
## $phenotypes
##    S/R fraction
## 1 SR_R   0.1875
## 2 SR_S   0.5625
## 3 SS_S   0.2500
```

```
match("Bb~cc | EE", BCEcombi) # position
20

## [1] 20

allcrossBCE[[20]]

## $genotypes
##          fraction SRtrait
## BB~cc | EE     0.25    SR_S
## Bb~cc | EE     0.50    SR_S
## bb~cc | EE     0.25    SS_S
##
## $phenotypes
##    S/R fraction
## 1 SR_S     0.75
## 2 SS_S     0.25
```

Linking the phenotype to the genotype

Tables S20 and S21: Markers developed around GWAS peaks and further used to genotype selfed offspring at the C and E loci. DMPR (*Daphnia magna* - *Pasteuria ramosa*) markers information. Map 2.4 corresponds to the currently available *D. magna* draft genome version 2.4 (Routtu et al. 2014). DMPR1 and 2 are physically linked to the C locus while DMPR3 and 4 are physically linked to the E locus. According to the genetic model underlying resistance to *P. ramosa* yielded from the GWAS analysis and previous studies (Metzger et al. 2016), resistance is dominant at the C locus (resistance to C1 and C19) whereas resistance is recessive at the E locus (resistance to P20). In addition, an epistatic relationship linking both loci confers susceptibility to P20 to an individual that shows susceptibility to C1 and C19, disregarding the individual genotype at the E locus. This model is described in Fig. 4. In individuals showing resistance to C1 and C19 (RR␣␣ phenotype, "Cc" or "CC" genotype), DMPR1 and 2 display either a heterozygous pattern with an allele linked to the C locus dominant allele (called R allele) and a recessive allele (S allele) ("Cc" genotype) or a homozygous pattern with one (or two: R1 and R2) dominant allele(s) ("CC" genotype). Note that at markers DMPR1, 3 and 4, two R alleles (called R1 and R2) are found, although R1 and R2 of DMPR4 have identical size. In individuals showing susceptibility to C1 and C19 (SS␣␣ phenotype, "cc" genotype), DMPR1 and 2 display a homozygous pattern with an allele linked to the C locus recessive allele (called S allele) ("cc" genotype). In individuals showing resistance to C1 and C19 (without epistatic relationship between C and E loci) and susceptibility to P20 (RR␣S phenotype, "Ee" or "EE" genotype at E locus), DMPR3 and 4 display either a heterozygous pattern with an allele linked to the E locus dominant allele (called S allele) and a recessive allele (R allele) ("Ee" genotype) or a homozygous pattern with an allele linked to the E locus dominant allele (called S allele) ("EE" genotype). In individuals showing resistance to C1 and C19 (without epistatic relationship between C and E loci) and resistance to P20 (RR␣R phenotype, "ee" genotype at E locus), DMPR3 and 4 display a homozygous pattern with an allele linked to the E locus recessive allele (called R allele) ("ee" genotype). In individuals susceptible to C1 and C19 (SS␣␣ phenotype), DMPR3 and 4 can show one of the three patterns described above (corresponding to "Ee", "EE" or "ee" genotype) because of the epistatic relationship linking the C and the E loci.

Table S20. DMPR (*Daphnia magna* - *Pasteuria ramosa*) markers information.

A: Description of the positions and sizes of the markers. All primers were used in the same master mix. The size of a marker corresponds to the size of the sequence from the beginning of the F-primer to the end of the R-primer on the reference genome (map 2.4): $size\ marker = \ end\ position - start\ position\ of\ the\ marker$.

B and C: Description of primers and motifs. Reference motifs correspond to the motifs that are present in the reference genome (map 2.4). The size of an amplicon is calculated as follows: $size\ amplicon = size\ of\ the\ marker - size\ of\ the\ reference\ motif + size\ of\ the\ motif\ of\ interest$. For example, to obtain the size of the R1 allele of DMPR1 we get: $size\ of\ DMPR_{R1}amplicon = size\ of DMPR1 - size\ of\ DMPR1\ reference\ motif + size\ of\ DMPR1_{R1}motif = 118 - 31 + 2 = 89$.

D: Expected marker signatures for all possible genotypes. Here we consider alleles as the observable peaks yielded by the marker analysis, as two copies of the same sequence in a homozygous individual will be observed as a single amplicon of a given size. A “resistance” (R) or “susceptibility” (S) allele does not necessarily confer resistance or susceptibility to the individual that carries it. Resistance phenotype will depend on the genetic model described above. For DMPR1, 3 and 4, we expect R homozygous individuals (“CC” or “ee” genotype) to present one of the two R alleles (R1 or R2) or both. Heterozygous individuals (“Cc” or “Ee” genotype) should present one R allele (R1 or R2) together with the S allele. Finally, S homozygous individuals (“cc” or “EE” genotype) should present only the S allele. Similarly, for DMPR2, we expect R homozygous individuals to present only the R allele (R1), heterozygous individuals to present the R allele (R1) together with the S allele and S homozygous individuals should present the S allele. Note that for DMPR4, because both R1 and R2 alleles have the same size, they will appear as a single peak during the marker analysis.

A

Name of marker	Concentration in master mix (µM)	Locus to which marker is physically linked	Scaffold (map 2.4)	Start position of the marker (map 2.4)	End position of the marker (map 2.4)	Size of the marker (bp) (map 2.4)	Position of the motif (map 2.4)
DMPR1	0.1	C	944	1350608	1350726	118	1350644
DMPR2	0.1	C	944	1563359	1563543	184	1563391
DMPR3	0.6	E	2167	19662	19874	212	19793
DMPR4	0.6	E	2560	81434	81570	136	81471

B

Name of marker	Sequence of forward (F) primer	F-Primer size (bp)	Sequence of reverse (R) primer	R-Primer size (bp)	Melting temperature (°C)	Reference motif (RM) (map 2.4)	RM length (bp)
DMPR1	ACAGCACGTCTCCGACTAAGG	21	GACGCCAAMAMCTACGCAACC	21	50	AAACGCACGGATCCTATATG TATCGAGCTTA	31
DMPR2	CAAATCTGCAATGGAATGAAAG	22	AACGCAACCGTTACGGTTAC	20	50	CTCCTGCTGGCT	12
DMPR3	TTACGTTCGGTTTGGCTCCG	20	TGAAACATTGGTAAGAGACG	20	48	TACAACAACAACAACAACAA CAA	23
DMPR4	GATAGATATTTATTGAACAG	20	TTTTGTCTTCGGAAGAACG	19	48	AATGCCTCCATGCCTCCATGC CTCCA	26

C

Name of marker	Motif of allele(s) conferring resistance (R1 and R2) (dominant at C locus and recessive at E locus)	Motif R1 length (bp)	Motif R2 length (bp)	Motif of allele conferring susceptibility (S) (recessive at C locus and dominant at E locus)	Motif S length (bp)	Size of R1 amplicon (bp)	Size of R2 amplicon (bp)	Size of S amplicon (bp)
DMPR1	AA; AAATGCATATGTATATATCGAGCTTA	2	26	AAACGCACGGATCCTATATGTATCGAG CTTA	31	**89**	**113**	**118**
DMPR2	ATCCTGCTGGCT	12	NA	ATCG	4	**184**	NA	**176**
DMPR3	TACAACAACAACAACAACAAAA ; TACAACAACAACAACAACAACAAA	22	23	TACAACAACAACAAAAA	17	**211**	**212**	**206**
DMPR4	AATGCCTCCATGCCTCCA ; AATACCTCCATGCCTCCA	18	18	AATGCCTCCATGCCTCCATGCCTCCA	26	**128**	**128**	**136**

D

Name of marker	Expected marker signature of R homozygous individual ("CC" or "ee" genotype)	Expected marker signature of heterozygous individual ("Cc" or "Ee" genotype)	Expected marker signature of S homozygous individual ("cc" or "EE" genotype)
DMPR1	R1 / R2 or R1 + R2	R1 + S or R2 + S	S
DMPR2	R1	R1 + S	S
DMPR3	R1 / R2 or R1 + R2	R1 + S or R2 + S	S
DMPR4	R1 / R2 or R1 + R2	R1 + S or R2 + S	S

Table S21. PCR reaction cycles using DMPR1 to 4.

Temperature	Time	Cycles
95 °C	15 min	
94 °C	30 sec	
60 °C	1 min 30 sec	30 x
72 °C	1 min 30 sec	
94 °C	30 sec	
47 °C	1 min 30 sec	10 x
72 °C	1 min 30 sec	
72 °C	10 min	
8 °C	∞	

References

Andras JP, Ebert D. 2013. A novel approach to parasite population genetics: Experimental infection reveals geographic differentiation, recombination and host-mediated population structure in *Pasteuria ramosa*, a bacterial parasite of *Daphnia*. *Mol. Ecol.* 22:972–986.

Bento G, Routtu J, Fields PD, Bourgeois Y, Du Pasquier L, Ebert D. 2017. The genetic basis of resistance and matching-allele interactions of a host-parasite system: The *Daphnia magna-Pasteuria ramosa* model. *PLOS Genet.* 13:e1006596.

Dukić M, Berner D, Roesti M, Haag CR, Ebert D. 2016. A high-density genetic map reveals variation in recombination rate across the genome of *Daphnia magna*. *BMC Genet.* 17:137.

Metzger CMJA, Luijckx P, Bento G, Mariadassou M, Ebert D. 2016. The Red Queen lives: Epistasis between linked resistance loci. *Evolution* 70:480–487.

Routtu J, Hall MD, Albere B, Beisel C, Bergeron RD, Chaturvedi A, Choi J-H, Colbourne J, De Meester L, Stephens MT, et al. 2014. An SNP-based second-generation genetic map of *Daphnia magna* and its application to QTL analysis of phenotypic traits. *BMC Genomics* 15:1–15.

Signorell A et al. 2018. DescTools: Tools for descriptive statistics. R package version 0.99.26.
